# Matrix metalloproteinase-19 inhibits growth of endothelial cells by generating angiostatin-like fragments from plasminogen

**DOI:** 10.1186/1471-2091-12-38

**Published:** 2011-07-25

**Authors:** Rena Brauer, Inken M Beck, Martin Roderfeld, Elke Roeb, Radislav Sedlacek

**Affiliations:** 1 Institute of Biochemistry, University of Kiel, Kiel, Germany; 2 Institute of Molecular Genetics, Prague, Czech Republic; 3 Institute of Biotechnology, Prague, Czech Republic; 4 Department of Gastroenterology, Medical Clinic II, Justus Liebig University, Giessen, Germany

## Abstract

**Background:**

Angiogenesis is the process of forming new blood vessels from existing ones and requires degradation of the vascular basement membrane and remodeling of extracellular matrix (ECM) in order to allow endothelial cells to migrate and invade into the surrounding tissue. Matrix metalloproteinases (MMPs) are considered to play a central role in the remodeling of basement membranes and ECM. However, MMPs contribute to vascular remodeling not only by degrading ECM components. Specific MMPs enhance angiogenesis via several ways; they help pericytes to detach from vessels undergoing angiogenesis, release ECM-bound angiogenic growth factors, expose cryptic pro-angiogenic integrin binding sites in the ECM, generate promigratory ECM component fragments, and cleave endothelial cell-cell adhesions. MMPs can also negatively influence the angiogenic process through generating endogenous angiogenesis inhibitors by proteolytic cleavage. Angiostatin, a proteolytic fragment of plasminogen, is one of the most potent antagonists of angiogenesis that inhibits migration and proliferation of endothelial cells. Reports have shown that metalloelastase, pancreas elastase, plasmin reductase, and plasmin convert plasminogen to angiostatin.

**Results:**

We report here that MMP-19 processes human plasminogen in a characteristic cleavage pattern to generate three angiostatin-like fragments with a molecular weight of 35, 38, and 42 kDa. These fragments released by MMP-19 significantly inhibited the proliferation of HMEC cells by 27% (p = 0.01) and reduced formation of capillary-like structures by 45% (p = 0.05) compared with control cells. As it is known that angiostatin blocks hepatocyte growth factor (HGF)-induced pro-angiogenic signaling in endothelial cells due to structural similarities to HGF, we have analyzed if the plasminogen fragments generated by MMP-19 interfere with this pathway. As it involves the activation of c-met, the receptor of HGF, we could show that MMP-19-dependent processing of plasminogen decreases the phosphorylation of c-met.

**Conclusion:**

Altogether, MMP-19 exhibits an anti-angiogenic effect on endothelial cells via generation of angiostatin-like fragments.

## Background

Angiogenesis is the process of formation of capillaries that sprout from existing blood vessels. It plays an essential role in several physiological processes such as wound healing, female reproduction, embryonic development, organ formation, and tissue regeneration and remodeling [1]. In pathological processes, the abnormal growth of new blood vessels can lead to the progression of many diseases including tumor growth.

Angiostatin is a potent angiogenesis inhibitor specific for endothelial cells. It is a single chain proteolytic fragment consisting of the first four triple disulfide-linked kringle domains of plasminogen [2,3]. Functional angiostatin-like molecules can be generated from plasmin reduction and proteolysis [4], plasminogen digestion by pancreas elastase [5], urokinase-activated plasmin [6], prostate specific antigen [7], cathepsin D [8], and by several matrix metalloproteinases, including MMP-12 [6,9], matrilysin or MMP-7, MMP-9 [9,10], and MMP-2 [9,11].

Matrix metalloproteinase-19 (MMP-19) was originally isolated from the inflamed synovium of a rheumatoid arthritis patient [12], from mammary gland, and liver [13,14]. Human and murine orthologues of MMP-19 (human: U37791, murine: AF153199) retain the common domain organization of soluble members of the MMP family, however, they also contain several distinctive features including a unique cysteine in the catalytic domain, an altered latency motif, a unique oligoglutamate insertion in the hinge region, and a C-terminal tail [12-17]. MMP-19 is expressed in many tissues at mRNA level [13,14] although its expression at protein level appears to be more restricted. Vascular smooth muscle cells, myoepithelial cells, and basal keratinocytes express MMP-19 constitutively whereas endothelial cells, epithelial cells of the mammary glands as well as monocytes and macrophages show differential regulation of this enzyme [18-23].

MMP-19 was reported to degrade several basement membrane proteins such as type IV collagen, laminin 5 γ2 chain, tenascin C, and nidogen-1 [17,24-26]. This capacity together with the expression pattern may point to a role of MMP-19 in vascular remodeling and angiogenesis. In the present study, we report that recombinant MMP-19 specifically generates angiostatin-like fragments from plasminogen, which inhibit proliferation and capillary-growth of endothelial cells.

## Results

### GST-MMP-19 processes Glu-type plasminogen to angiostatin-like fragments

**Figure 1:**
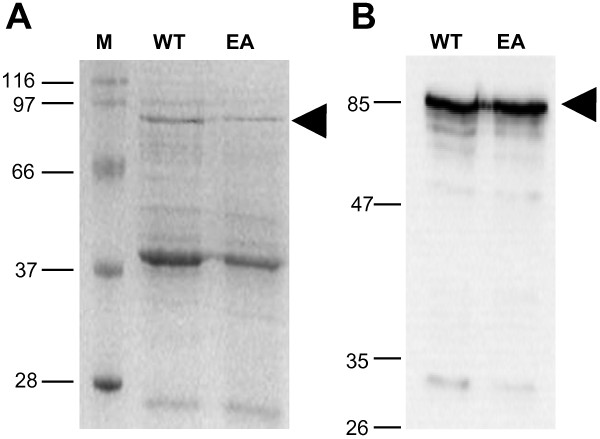
**Purification and detection of recombinant GST-MMP-19 proteins**. (A) 5 μg purified active MMP-19 protein (WT) and the mutant inactive form of MMP-19 harbouring the exchange of E213 to A (EA) were subjected to SDS-PAGE and stained with silver staining. (B) Purified MMP-19 WT and EA protein (5 μg) were detected by Western blotting using rabbit polyclonal antibodies (Dianova, dilution 1:3000). Arrowheads indicate MMP-19 at 85 kDa.

To assess if plasminogen is a substrate of MMP-19, we used two types of the protein, Glu- and Lys-type plasminogen. Whereas the Glu-variant is the native form of the protein, the Lys-variant is generated by cleavage of the peptide bond between Lys77 and Lys78 by plasmin. In contrast to the Glu-type plasminogen, we observed self-degradation of the Lys-type form, even in the presence of the serine protease inhibitor aprotinin. Thus, we decided to continue the experiments with the Glu-type variant, which does not have any plasmin activity and nearly no self-degradation. As controls, we used samples with MMP inhibitor (MMP-9/MMP-13 inhibitor II) or the inactive MMP-19 mutant (E213A) instead of the wild-type fusion protein. The MMP-19 fusion protein was generated and purified as described in "Methods". The expected size of the purified fusion protein was 85 kDa as detected by Coomassie staining and immunoblotting using anti-MMP-19 antibody (Figure 1, arrowhead). The strong protein band of approximately 40 kDa appearing in the Coomassie stained SDS-PAGE is a peptide composed of the N-terminal GST-tag and the propeptide domain of MMP-19, which is generated during purification due to autocatalytic activity of MMP-19.

**Figure 2:**
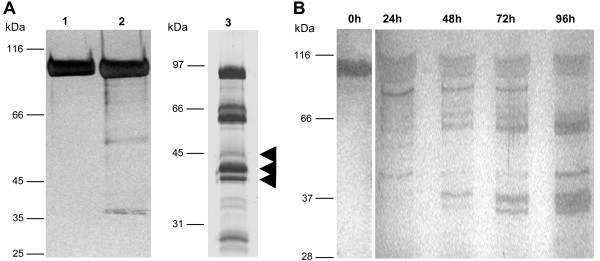
**Processing of human plasminogen by GST-MMP-19**. (A) 20 μg Glu-type plasminogen was either left undigested (lane 1) or was digested for 60 h by 5 μg MMP-9 (lane 2) which was activated before the digest with trypsin at 37°C for 20 min or 15 μg GST-MMP-19 (lane 3) at 37°C for 96 h. Digests were subjected to SDS-PAGE followed by Coomassie staining. Angiostatin-like fragments generated by MMP-19 are indicated by arrowheads. (B) Time course of human plasminogen processing by GST-MMP-19. 10 μg Plasminogen was digested by 8 μg GST-MMP-19 at 37°C for 96 h. Samples were analyzed at 0, 24, 48, 72, and 96 h on SDS-PAGE followed by silver staining. The mixture of digested plasminogen fragments was used without further purification in the tube-like formation assay.

We also used recombinant murine MMP-9 in an initial experiment as it was published that MMP-9 generates angiostatin like fragments [10]. The same experimental conditions were applied to both MMPs to be able to compare their efficiency of both MMPs. The processing of plasminogen by MMP-9 was not as efficient as the one of MMP-19, thus, it was not included in the following experiments (Figure 2A).

Processing of human Glu-type plasminogen by MMP-19 for 96 h generates several fragments with an apparent molecular weight of 35 (kringle 1-3), 38 (kringle 1-4), and 42 kDa (Figure 2A), some of them correspond to the angiostatin-like fragment (38 kDa). The protein band about 92 kDa represents the full-length Glu-type plasminogen. As the angiostatin-like fragments consist of the N-terminal part of plasminogen the bands below 66 kDa are the corresponding C-terminal parts of the cleaved protein. Comparing the time course of this reaction, an increase of the putative angiostatin-like fragments over time was obvious (Figure 2B). Fragments that occurred and disappeared during the incubation are intermediate products that are further processed to the final pattern of fragments (Figure 2B).

### Plasminogen processed by GST-MMP-19 inhibits proliferation of microvascular endothelial cells

**Figure 3:**
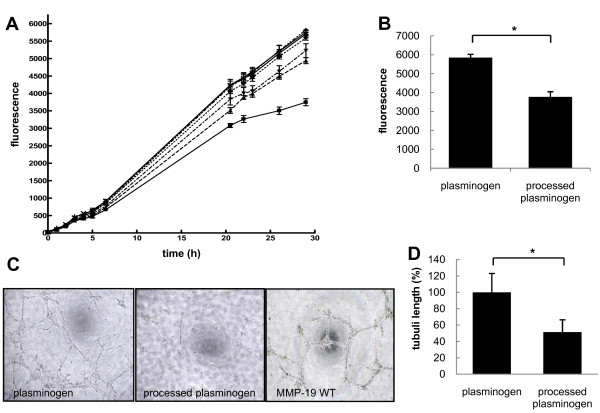
**Effect of plasminogen processing on cell proliferation and angiogenesis**. (A) Proliferation of microvascular endothelial cells HMEC-1 is inhibited by GST-MMP-19 processed plasminogen. Plates were coated as follows: black diamond, plasminogen; black circle, aprotinin; black square, processed plasminogen; black triangle, processed plasminogen with MMP inhibitor; inverted black triangle, MMP inhibitor; + (plus symbol), GST-MMP-19 wild-type; cross, GST-MMP-19 inactive mutant. Proliferation was measured over 30 h with 2500 HMEC-1 cells per well and using the Alamar Blue assay. Standard deviation was calculated from triplicates. (B) Proliferation in response to processed plasminogen compared to non-processed plasminogen at assay endpoint (30 h); * p = 0.01 (C) *In vitro *angiogenesis assay. Processed plasminogen inhibits capillary-like structure formation of HMEC-1 cells (D) The length of tubuli-like structures was measured in at least three wells per experiment. The mean ± SD of the tubuli length is given as the percentage of the control. * p = 0.05.

To investigate anti-angiogenic properties of generated plasminogen fragments, we first analyzed their effect on proliferation of HMEC-1 endothelial cells using the Alamar Blue proliferation assay (Biosource). Cell proliferation started to decrease after 10 h (Figure 3A) in the presence of the processed plasminogen and was reduced about 27% compared to full-length plasminogen after 30 h (Figure 3B). This proliferation decrease appears to be due to the presence of active angiostatin-like fragments in the digested plasminogen mixture. Recombinant MMP-19 (2 μg/well) alone does not show any inhibition of the proliferation.

### Capillary-like formation is inhibited by fragments generated by MMP-19

To assess the effect of plasminogen fragments generated by MMP-19 on endothelial cell differentiation, *in vitro *angiogenesis assays were performed. HMEC-1 cells were placed on Matrigel in EGM-MV medium containing recombinant MMP-19 (1 μg/well), plasminogen (1.25 μg/well), or cleavage products of plasminogen. The cells were examined after 24 h of incubation regarding the induction of formation of tube-like structures (Figure 3C). The tube formation of the cells treated with the processed plasminogen mixture was remarkably reduced in comparison to the control. The amount of capillary-like structures was about 47% less in the treated than in control wells (set to 100%; Figure 3D).

### Plasminogen processed by GST-MMP-19 decreases the phosphorylation of c-Met and Akt/PKB

**Figure 4:**
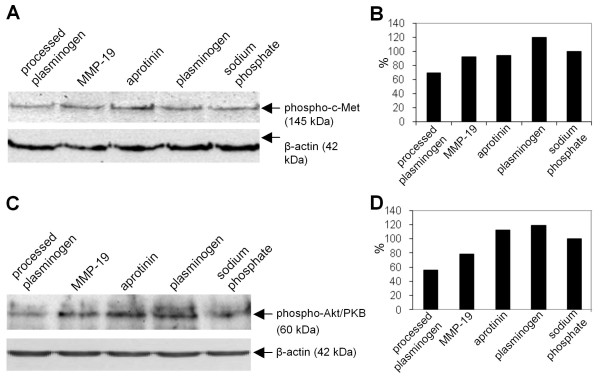
**Angiostatin inhibits phosphorylation of c-Met and Akt/PKB in HMEC-1 cells**. HMEC-1 cells were incubated at 37°C and 5% CO_2 _for 40 h. For the treatment with plaminogen mixture, 2 μg/well recombinant MMP-19, or aprotinin 1 ug/ml were used. Cell lysates were prepared and analyzed by 12.5% SDS-PAGE followed by immunoblotting for phospho-cMet (A) and phospho-Akt/PKB (B). Blots were then stripped and re-probed for β-actin as a loading control. Quantification of phospho-cMet and phospho-Akt/PKB protein was performed by densiometric analysis using the Aida Image Analyser and normalized for the signal intensity of the beta-actin band. Densitometric analysis; intensity of phospho-c-Met (B) and phospho-Akt/PKB (D) bands was normalized to the corresponding signal intensity of β-actin bands and the buffer control was set as 100%. Sodium phosphate, used as buffer for the above experiments, was used as a control. One of two independent experiments is shown.

Because angiostatin blocks HGF-induced angiogenesis by inhibition of phosphorylation of its cell surface receptor c-Met, we analyzed whether the angiostatin-like fragments generated by MMP-19 mediated the described effects [27,28]. As demonstrated by Western blot analysis, phosphorylation of c-Met in HMEC-1 cells was inhibited by 27% after treatment with processed plasminogen compared to plasminogen (Figure 4A and 4B). We further analyzed phosphorylation of Akt/PKB after treatment with the angiostatin-like fragments. Apart from c-Met, Akt signaling is also essential in angiogenesis as it affects the cell cycle and therefore proliferation. We observed a decrease of 45% of the phosphorylated form of Akt kinase after treatment with processed plasminogen compared to plasminogen (Figure 4C and 4D).

## Discussion

Plasminogen, a single-chain glycoprotein of 92 kDa consisting of an N-terminal peptide, five kringle domains, and a serine protease domain [29], plays a crucial role in tumor metastasis and angiogenesis where localized proteolysis is required. Under certain conditions, plasminogen undergoes proteolysis to form kringle-containing A-chain fragments, collectively called angiostatins [2,30,31], which are novel and potent inhibitors of endothelial cell proliferation and tumor angiogenesis [9]. Typically, angiostatin consists of the first four kringle domains (K1-4). Plasminogen is cleaved by several proteases, among them members of matrix metalloproteinase family, that are derived from tumor cells or infiltrating macrophages [8,32]. These kringle domains and their relatives inhibit the proliferation of vascular endothelial cells, a fundamental process in angiogenesis [31].

As MMP-19 was reported to be expressed by endothelial cells as well as cells that surround endothelium and capillaries, it could be expected that its activity likely impacts vascular processes including angiogenesis. Our experiments show that MMP-19 has angiostatin-converting enzyme activity and generates angiostatin-like fragments similar to MMP-3, -7, -9, and -12 [9-11]. The cleavage site is located between kringle 5 and the protease domain to generate angiostatin molecules consisting of all five kringle domains of plasminogen. As all reported angiostatin species exhibit the biological activities of angiostatin isolated from plasma (e.g. inhibition of endothelial proliferation, angiogenesis, and tumor growth and metastasis), the angiostatin-like fragments generated by MMP-19 should also have such biological activities.

Angiogenic growth factors and inflammatory cytokines can induce a number of pericellular acting proteases, including MT1-MMP, MMP-2, MMP-9, and u-PA. This is generally seen as a part of the repertoire of cellular activities that are switched on when the pro-angiogenic growth factors overrule the angiogenesis-inhibiting factors. However, this unidirectional view on the relation between angiogenic growth factors and proteases has evolved into the insight that proteases themselves also contribute to fine-tuning of the activities of various growth factors that control the onset and progression of angiogenesis [33-35]. Different members of the MMP family may generate angiostatin-like fragments with different efficiency and with different composition. The contribution of MMP-19 and other MMPs to angiostatin generation *in vivo *will depend on their expression pattern, the rate of their activity, and also inactivation by endogenous inhibitors. This mechanism may be further complicated by interactions between different MMPs as well as by multiple proteolytic activities towards extracellular matrix proteins in basement membrane or vascular bed. Thus, MMP-19 could further increase its anti-angiogenic effect by damage of basement membrane scaffold that supports differentiation processes of endothelial cells. MMP-19 could achieve this by cleavage of at least three important basement membrane components: tenascin C, γ2chain of laminin5, and nidogen-1 [24-26]. In our previous study we could show that high concentrations of MMP-19 might have negative influence on endothelial cell growth as MMP-19-dependent processing of nidogen-1 led to inhibition of tube-like formation *in vitro *[24]. As higher concentrations of MMP-19 could influence or interfere with effects of processed plasminogen we tested the remaining MMP-19 fusion proteins in the processed plasminogen mixture on endothelial cells as well. However, MMP-19 under these experimental conditions did not exhibit any effect on the cells.

Moreover, current data show that MMP-19 exhibit also critical antitumor activity as secreted active MMP-19, but not the inactive mutant, induces reduction of tube-forming ability in endothelial cells with decreased vascular endothelial growth factor (VEGF). Thus, MMP-19 seems to be responsible, at least partly, for bioavailability of MMP-2 and VEGF that promote angiogenesis [36]. In contrast, the MMP-19-deficient mice showed decreased tumor angiogenesis and invasion [37] pointing, thus, to a potential dual role of MMP-19. The pro-angiogenic role of MMP-19 could be associated with its expression in microvascular endothelial cells or smooth muscle cells [19], and in the controlled release of pro-angiogenic factors such as VEGF and MMP-2; the anti-angiogenic effect of MMP-19 might originate from uncontrolled overproduction of this MMP from various surrounding cellular sources, which can disrupt the necessary ECM scaffold or, as here reported, produce angiostatin-like fragments.

As MMP-19 generates angiostatin-like fragments that subsequently inhibit endothelial cell proliferation and tube-like formation, we asked, which pathways are involved in this inhibition. c-Met is the HGF receptor that controls cellular mobility due to tyrosine-kinase activity. HGF binding to its receptor induces the tyrosine autophosphorylation of the receptor catalytic domain that initiates the intracellular signaling. Angiostatin has structural similarities to HGF that promotes angiogenesis, induces proliferation, migration, and also influences cell survival via its cell surface receptor, c-Met. Upon HGF stimulation, c-Met induces several biological responses that collectively give rise to a program known as invasive growth. It is thought that angiostatin inhibits HGF-induced phosphorylation of c-Met, Akt, and ERK1/2 via binding to soluble c-Met. Angiostatin and c-Met form a stable complex and affect signaling events induced by HGF but not by VEGF or bFGF [27].

The inhibition of Akt phosphorylation by angiostatin is not solely a marker for the inhibition of HGF binding to c-met; instead, a reduction in phospho-Akt could directly contribute to the disruption of angiogenesis. Akt is a serine/threonine kinase that is rapidly activated as a downstream effector of phosphatidylinositol 3 (PI3) kinase in response to a variety of cytokines and growth factors, including HGF [38]. In this work we could show that MMP-19-processed plasminogen inhibits the HGF-induced phosphorylation of c-Met and Akt/PKB and that plasminogen fragments generated by MMP-19 impact proliferation and tube-like formation of endothelial cells.

## Conclusion

We report here that MMP-19 processes human plasminogen and generates angiostatin-like fragments that inhibit proliferation microvascular endothelial cells, decreases the phosphorylation of c-met, and reduce formation of capillary-like structures. Thus, MMP-19 exhibits an anti-angiogenic effect on endothelial cells via generation of angiostatin-like fragments.

## Methods

### Expression and purification of human MMP-19 GST-fusion protein (GST-MMP-19)

MMP-19 was produced as a fusion protein with glutathione-S-transferase (GST) in the BLR (DE3) strain of *E. coli *(Novagen, Darmstadt, Germany) using the expression vector pGEX-2T. The recombinant protein starts N-terminally with the GST fused in frame to Phe, the first amino acid of the propeptide domain, and ends with Arg, the first amino acid of the 36 amino acid-long C-terminal tail. The expression of MMP-19 was induced by 0.6 mM Isopropyl-1-thio-D-galactopyranoside (IPTG). MMP-19 was produced as a fusion protein of glutathion-S-transferase (GST) and MMP-19 as described [23,25]. Purification was done according to Rohman and Harrison-Lavoie [39] with slight modifications. In brief, the pelleted bacteria were resuspended in 20 ml buffer A (sonication buffer) (100 mM Triethanolamine-HCl (TEA-HCl), 150 mM NaCl, 1% Triton X-100, pH 7.4) and disrupted in the presence of Complete™proteinase inhibitor (Roche, Mannheim, Germany) by sonification. The sonicate was pelleted and the supernatant transferred into 4 ml of buffer B (100 mM TEA-HCl, 200 mM MgCl_2_, 500 mM KCl, 100 mM ATP, pH 7.4) and incubated for 30 min at room temperature. This step was followed by an incubation for 45 min with 0.5 ml 50% slurry of Glutathione Sepharose 4B (GE Healthcare, Freiburg, Germany). The gel was washed three times with 10 ml buffer C (100 mM TEA-HCl, 150 mM NaCl, 20 mM MgCl_2_, 50 mM KCl, 1% Triton, 10 mM ATP, protease inhibitors, pH 7.4). In the last washing step buffer D was used (100 mM TEA-HCl, 150 mM NaCl, 20 mM MgCl_2_, 50 mM KCl, 5 mM ATP, pH 7.4). For elution of the bound fusion protein we used 50 mM Tris-HCl with 10 mM reduced glutathione, pH 8.0 which is prepared freshly prior use. We performed 5 elutions and analyzed them by SDS-PAGE. The fractions were pooled and dialysed over night at 4°C against 2 l TNC buffer (50 mM Tris-HCl, 150 mM NaCl, 5 mM MgCl_2_, 5 mM CaCl_2_, pH 7.4) using a Slide-a-lyser cassette (PIERCE, Ulm, Germany) to get rid of the reduced glutathione. The concentration was determined using BCA kit (PIERCE, Ulm, Germany).

Immunoblotting for MMP-19 was performed using a rabbit polyclonal antibody (Dianova, dilution 1:3000) against the hinge region of MMP-19. This antibody detected the zymogen, the active protein as well as wild-type (WT) and inactive mutant (EA).

The murine proMMP-9 protein as a control was expressed in Cos7 cells. The protein was purified by affinity chromatography binding to a gelatine sepharose column (GE Healthcare, Freiburg, Germany). Before using recombinant MMP-9 in the cleavage assay it has to be activated with trypsin (10 μg/ml) at 37°C for 20 min. The reaction was stopped by adding trypsin inhibitor (1 mg/ml).

### Preparation of proteolytic fragments of plasminogen and analysis

The processing of 10 μg plasminogen (human Glu-type plasminogen, Calbiochem or Lys-type plasminogen, Chromogenix) was done in TNC buffer + 10 μM ZnCl_2_, pH 7.4 with 50 μl GST-MMP-19 (8 μg) at 37°C for 96 h; samples were taken every 24 h. To determine the specificity we used the following controls: One control was without any enzyme to observe the self-processing. Second control was the use of GST-MMP-19 inactive mutant (E213A) instead of active protein. Third control contained an MMP-19 inhibitor (10 nM; MMP-9/MMP-13 inhibitor II, Calbiochem), which was chosen because of the strong inhibition of recombinant human MMP-19 (data not shown). To avoid the activation and the autocatalytic activity of the zymogene plasminogen to its active form plasmin (a serine protease), we used serine protease inhibitor Aprotinin (1 μg/ml; Sigma, Germany). Also a control without Aprotinin was analyzed. To compare the efficiency of the cleavage to other MMPs 10 μg plasminogen (human Glu-type plasminogen, Calbiochem) was incubated with 5 μg recombinant MMP-9 using the same experimental conditions. proMMP-9 was activated prior to trypsin treatment at 37°C for 20 min. The mixture of digested plasminogen fragments was used without further purification in the tube-like formation assay.

### Cell culture

Human microvascular endothelial cells (HMECs), kindly provided by Prof. Marmé (Freiburg, Germany), were cultured in Endothelial Cell Growth Medium MV with Supplement Mix (EGM-MV) (Promocell, Heidelberg, Germany) in a humidified atmosphere of 5% CO_2 _at 37°C.

### Endothelial cell proliferation assay

A 96-well flat bottom plate was coated with GST-MMP-19 processed plasminogen or the following controls: unprocessed plasminogen (2.5 μg/well), GST-MMP-19 WT or EA (2.0 μg/well), GST-MMP-19 with inhibitor, aprotinin, or TNC buffer. An uncoated plate served as additional control. HMECs were then added (2500 cells/well) and the plate incubated at 37°C with 5% CO_2_. To evaluate the effect of the processed Glu-type plasminogen on cell proliferation, we used the Alamar Blue colorimetric assay (Biosource, Solingen, Germany) according to the manufacturer's instructions.

### Immunoblotting

HMEC-1 cells, were grown for 40 h in EGM-MV (Promocell) supplemented with reaction buffer (sodium phosphate) alone, with aprotinin (1 μg/ml), with MMP-19 (2 μg/well), or with processed and unprocessed plasminogen as described above. Cell lysates were prepared as described previously and 40 μg protein per sample was applied to SDS-PAGE; anti-phosphorylated c-Met (Biosource, dilution 1:500 in TBS + 1% BSA) or anti-phosphorylated Akt/PKB (Biosource, dilution 1:500 in TBS + 1% BSA) were used for detection. Bound antibody was detected using peroxidase-conjugated anti-rabbit antibody (PIERCE, USA) and the ECL plus Western Blotting Detection System (GE Healthcare, UK). Signals were recorded with a Luminescent Image Analyzer (LAS-3000, Fujifilm Life Science, USA) and analyzed with AIDA image analysis software (Raytest, Straubenhardt, Germany). Densitometric scans of the signal intensity of phospho-c-Met and phosphor-Akt/PKB bands are normalized for the corresponding signal intensity of β-actin bands; the buffer control was set as 100%.

### Tube-like formation assay *in vitro*

To evaluate the angiogenic effects of the plasminogen fragments *in vitro*, a tube-like formation assay was adapted from *Kubota et al*. [40] and *Donovan et al *[41]. Shortly, Matrigel (BD Bioscience, Heidelberg, Germany) was coated according to the manufacturer's protocol. The inner well of angiogenesis plates (μ-Slide Angiogenesis, ibidi, Martinsried, Germany), were filled with 10 μl Matrigel. The plate was incubated for 37°C to allow the Matrigel to gel and 5000 HMEC-1 cells per well were seeded onto the matrix. Images were captured using a digital camera and Olympus IX 51 microscope. Capillary-like formation, i.e. tubuli length, was evaluated after 24 h of incubation at 37°C. Two independent experiments were conducted and at least 3 wells evaluated per experiment. The total length of tubuli was measured in pixels using the image analysis software imageJ (NIH, US). The tubuli length is given as mean percentage of the control (± SD). To study the effect of the processed plasminogen the fragments were added to HMEC-1 cells. The same amount of non-processed plasminogen (1.25 μg/well) and recombinant MMP-19 (1 μg/well) was used as controls.

## Abbreviations

MMP: matrix metalloproteinase; GST: glutathione-S-transferase; HMECs: human microvascular endothelial cells; HGF: hepatocyte growth factor; IPTG: isopropyl-1-thio-β-D-galactopyranoside; VEGF: vascular endothelial growth factor; bFGF: basic fibroblast growth factor.

## Authors' contributions

RB carried out most of the experimental work and helped to draft the manuscript. IB participated in cell culture studies. MR and ER prepared recombinant MMP-9. RS made substantial contributions to conception, design, and coordination; prepared the manuscript. All authors read and approved the final manuscript.
